# Allocation of Credit Resources and “Borrow to Lend” Activities: Evidence From Chinese-Listed Companies

**DOI:** 10.3389/fpsyg.2022.856056

**Published:** 2022-03-31

**Authors:** Shangmei Zhao, Huibo Wang, Wei Li

**Affiliations:** School of Economics and Management, Beihang University, Beijing, China

**Keywords:** Borrow to Lend, monetary policy, credit rationing, credit equity, efficiency

## Abstract

Credit distribution is uneven in the domestic financial market since it is relatively easy for listed companies, mainly state-owned enterprises, to obtain banks’ funds. Unbalanced credit distribution has caused some listed companies to participate in “Borrow to Lend” activities. Based on the traditional “financing priority” theory and credit rationing theory, this paper studies the “Borrow to Lend” shadow banking activities of China’s non-financial listed companies based on the 2007–2018 financial statement data of Chinese-listed companies and discusses the micro-level and macro-level related factors behind this activity. The empirical results show that China’s non-financial listed companies, especially the state-owned enterprises, are participating in obvious “Borrow to Lend” activities. The real economy’s rate of return shows a negative relationship with “Borrow to Lend” activities at the level of individual companies and their industries. This article uses the exogenous growth part of M2 growth to measure monetary policy tightness in terms of macro and credit policies. It uses the ratio of state-owned enterprise loans to total corporate loans as an approximate indicator of the credit distribution structure. The empirical results indicate that state-owned enterprises’ “Borrow to Lend” activities have shifted in the same direction as the tightening of monetary policy after the financial crisis. The proportion of state-owned enterprise loans positively correlates with state-owned enterprises’ “Borrow to Lend” activities.

## Introduction

It is vital for countries where banks dominate the financial market to pay attention to the efficiency of credit resource allocation, as improper credit allocation will cause the economy to fall out of reality, severely restricting technological innovation and efficiency development. At present, China’s credit distribution situation is still very unbalanced, and enterprises are facing different financing constraints. On the one hand, the banking industry is dominated by state-owned commercial banks. Direct financing channels such as the stock and bond markets are tilted toward the state-owned economy, favoring large-scale enterprises. Thus, many financial resources flow to state-owned enterprises and large listed companies.

On the other hand, due to the lack of collateral and the excessive risks, the financing needs of private enterprises and small/medium-sized enterprises cannot be met. That is, they are facing severe credit constraints. SMEs play an integral role in constructing the modern economic system. The high economic development is vital for accelerating the country’s financial activities. Indeed, the current study shows that the biggest obstacle in SME’s establishment is the growing financial constraints (i.e., credit risk) that require proper management (Yadi et al., 2019). Credit constraint fundamentally causes an economic downturn, thus causing the institutions to fail.

In recent years, China’s small/medium-sized private enterprises have encountered dual credit rationing constraints from the public ownership of the banking industry and the market. It shows obvious characteristics of “financing difficulties,” and the phenomenon of enterprises with sufficient credit resources acting as financial intermediaries has appeared in the financial market.

Due to severe credit constraints, the phenomenon of lending outside the formal banking system has always existed in different forms. These innovative informal financial channels have formed a large-scale shadow banking system. According to the social financing data released by the People’s Bank of China, shadow banking is defined as the sum of entrusted loans, trust loans, and unaccepted bank drafts in the narrowest terms. Since 2007, China’s shadow banking has continued to grow rapidly at an annual rate of over 20%. Its scale increased from 2.5 trillion yuan in 2007 to 25 trillion yuan in 2017. In addition, according to the China Shadow Banking Report released by Moody’s, until 2016, the scale of China’s shadow banking accounted for about 82% of GDP during the same period, and two-thirds of shadow banking activities were loans through informal banking channels.

Under the current situation of unbalanced domestic credit distribution, in particular, many non-financial companies with relatively easy financing are engaged in substantial financial intermediary activities to provide funds for companies with financing difficulties to ease their credit constraints. The intermediary financial activities of these non-financial companies are the focus of this project. Under China’s existing financial system, interest rates have not been fully market-oriented. Major banks tend to lend to large state-owned enterprises and listed companies because of their sufficient collateral and low project default risk. Although some small/medium-sized enterprises implement investment projects with higher rates of return, they have difficulty financing through formal bank channels due to a lack of sufficient collateral; they can only switch to other non-bank financing channels. In addition to risk factors affecting credit decision-making, the credit market prefers state-owned enterprises and more substantial financing constraints on non-state-owned enterprises. As the state-owned enterprises bear the additional policy burden, the government will subsidize the state-owned enterprises through credit support. State-owned banks often lend to the state-owned enterprises due to policy considerations, making it easier for the state-owned enterprises to obtain credit support. Especially when the money supply is tightening, private enterprises’ debt growth rate slows down significantly, while state-owned enterprises still maintain rapid growth.

Under this circumstance, the phenomenon of enterprises with abundant credit resources playing an intermediary role and participating in lending emerged. More and more state-owned enterprises and listed companies have re-lent to private enterprises experiencing financing difficulties after obtaining bank loans or other financings. This article refers to this type of phenomenon as “Borrow to Lend.” These lending companies play the role of a financial intermediary, which can increase the social financing scale when monetary policy is tightened and partially offsets the decline in bank credit. In this context, whether the macro policy to regulate credit supply, especially the monetary policy, effectively regulates the credit resources flowing into the real economy and its mechanism of action has become an issue of widespread concern among academics, businesses, and governments.

In addition, since the beginning of 2013, the China Banking Regulatory Commission began to implement Basel III for commercial banks. The main features of Basel III are: First, it inherits and enriches the core capital regulatory standards of Basel I and II, expands the scope of capital coverage risks, and requires banks to implement comprehensive risk management based on risk quantification. Its second aim is to clarify multi-level capital supervision and ensure higher qualified capital standards. It requires the improvement of internal capital evaluation procedures, the establishment of a capital replenishment mechanism, and the continuous maintenance of capital adequacy. The third aspect is to take into account both macro-prudential and micro-prudential supervision. It is necessary to manage bank business risks and effectively resist the impact of systemic risks, carry out stress tests, and effectively respond to business cycle fluctuations. A major impact of the implementation of the agreement is that under stricter risk supervision, banks will allocate more credit resources to state-owned enterprises with lower risks and higher credit ratings due to the existence of government guarantees. This makes the initial allocation of corporate credit by commercial banks more unbalanced. Given the statement, the study suggests that to minimize the consequence of China’s policy burden, the government department should ensure the efficiency of the credit risk allocation, thus optimizing the Chinese financial system (Ye et al., 2021). However, Low-risk state-owned enterprises are more likely to use the funds as financial intermediaries for activities other than their entity investment after receiving a large amount of credit funds. This makes the strengthening of bank risk management indirectly important for corporate financial intermediary activities and the effectiveness of credit control policies. Accordingly, the study suggests adopting risk management strategies, thereby managing the credit risk (Rehman et al., 2019).

The “Borrow to Lend” activities of non-financial listed companies studied in this article do not include entrusted loans with own funds and do not include short-term cash surplus management before investment of credit funds. It is also different between subsidiaries of the same parent company or between subsidiaries and parent companies. Loans to related parties between companies are purely a credit intermediary act for earning interest margins and deviating from reality. Since it is not possible to directly observe the participation of non-financial companies in lending activities, this article is based on the existing literature. With the research of Shin and Zhao (2013) and Qi et al. (2021), identifying the “Borrow to Lend” financial intermediary activities of non-financial enterprises has been improved through the research of key financial data information enterprises. Also, based on the identification of “Borrow to Lend,” focusing on corporate micro, industry, and macro-financial perspectives, research on the relationship between the key factors such as the rate of return of the real corporate economy, macro-level monetary policy, the proportion of state-owned enterprises in non-financial corporate bank loans, and the “Borrow to Lend” activities of non-financial enterprises provides important empirical evidence for understanding the interrelationships between “Borrow to Lend” activities, monetary policy, and the proportion of state-owned enterprise bank loans. This article also provides new evidence regarding the characteristics of intermediary credit activities that follow the credit cycle, such as “Borrow to Lend” activities, which has strong policy significance for promoting the fairness and efficiency of credit resource allocation and curbing the real economy from removing from reality to virtual.

The structure of this article is as follows: Part 2 is a literature review and introduces the main contributions of this article. Part 3 establishes the identification model and related theoretical basis of “Borrow to Lend” and gives a concise introduction to the data plus an empirical analysis model of the influence of monetary policy and the proportion of state-owned enterprise bank loans on “Borrow to Lend” activities. The fourth part is the empirical results and the corresponding explanations, while the fifth part offers the conclusion and the outlook of the full text.

## Literature Review

Since the global financial crisis in 2008, academia has conducted extensive in-depth research on shadow banking systems in developed economies (Gorton and Metrick, 2012; Gennaioli et al., 2013), with studies focusing mainly on how the shadow banking system acts as a financial intermediary and its role in the financial market, and research objects including money market mutual funds, asset-backed securities, and other different forms. Also discussed are the risks that various shadow banking activities would cause to the financial system (Acharya et al., 2013; Kacperczyk and Schnabl, 2013; Krishnamurthy et al., 2014).

In recent years, the rapid expansion of China’s shadow banking has gained considerable attention, potentially influencing China’s economic development (Jiang and Fang, 2021). The literature introduces the overall situation regarding China’s shadow banking activities. Dang et al. (2014) compared the shadow banking systems of China and the United States. The key difference is that China’s shadow banking system is centered on traditional banking (Fève et al., 2019), so shadow banking activities have more contact with traditional banks. Li (2014) also found that China’s shadow banking is closely related to traditional banks and summarized the regulatory issues related to shadow banking activities. Hachem and Song (2015) studied how regulatory factors in the banking industry led to the rise of shadow banking activities.

Indeed, the Chinese shadow banking industry significantly supports the management of large transactions between banking institutions and non-financial firms, thus drastically influencing the on-balance-sheet allocation (Liao, 2020). Chen et al. (2018) used two micro-data sets of bank-level entrusted loans and off-balance sheet business investment. They found that non-state-owned banks, compared with state-owned banks, actively participate in shadow banking activities for off-balance-sheet business lending in response to monetary policy tightening. In addition, the rapid growth of China’s shadow banking in recent years has been driven by both demand and supply factors. Shadow banking in China plays a critical role in generating credit, thus hiding credit discrimination (Sun, 2019). On the one hand, due to limited collateral and the lack of political support, a considerable number of high-productivity companies suffer from credit discrimination; on the other hand, underdeveloped financial markets lack reliable investment tools (Song et al., 2011; Sarfraz et al., 2019).

In recent literature, researchers have begun to pay attention to several specific forms of shadow banking activities in China. Their research objects include Wealth Management Product (WMP), entrusted loans, trust business, etc. Acharya et al. (2021) studied the characteristics of wealth management products (WMP) in shadow banking activities and explored the impact of interest rate policies and bank supervision on the development of wealth management products (WMP). In addition, they also found that after the economic stimulus plan, China’s small/medium-sized banks have significantly increased shadow banking activities through the release of more wealth management products (WMP). In support, the study shows that shadow banking in China has immensely grown through the use of wealth management products, thus evading regulatory constraints (Shah et al., 2021). In particular, the entrusted loan is the most fundamental activity of shadow banking in China. Accordingly, China’s non-state enterprises provide financial support to the other party, thus influencing the overall company’s financial status (Wang et al., 2021).

Allen et al. (2019) contend that some companies with financing privileges tend to provide more loans to non-financial companies with financing difficulties in the form of entrusted loans. In addition, they also found that entrusted loans will increase correspondingly when credit is tightened. Chen et al. (2018) also explored the connection between China’s monetary policy tightening and entrusted loans and found that the monetary policy tightening will cause non-state banks to issue more entrusted loans.

These papers above focus on the behavior of traditional banks and discuss various shadow banking activities (wealth management products or entrusted loans). In addition, some previous studies analyze the topic from the perspective of inter-enterprise loans. He et al. (2016) studied the stock price reaction of two borrowers and lenders after the listed company announced inter-enterprise loans. The research results show that the average abnormal return of intercompany loan issuers is negative, while the receiver’s stock price has a positive response. The impact of corporate participation in financial activities on the real economy has also been verified in the literature. Zhang and Zhang (2016) pointed out that non-financial companies gradually show a financial trend in asset allocation and profit accumulation. The manufacturing industry will have more resources and energy concentrating on speculative activities in financial assets and productive investment, technological innovation activities have been suppressed, and the trend of economic “removal from reality to virtuality” is increasing. Zhang (2019) explained the logic of financialization based on the three levels of macro, micro, and meso. He pointed out that the “macro-financial development theory” and “micro-financial market theory” promoted the financialization of non-financial industries such as manufacturing and influenced the operation philosophy of micro-enterprises. This, coupled with the fact that companies seek profit and avoid risks in practice, drives the financialization of micro-enterprises.

In recent literature, based on corporate financial data, the identification of “Borrow to Lend” and its relationship with monetary policy and changes in the proportion of state-owned enterprise loans are studied from the perspectives of system and market. Few studies have revealed the micro-mechanism of credit fairness and their impact on macroscopic effects. Shin and Zhao (2013) found that large non-financial companies in India and China behave like financial intermediaries rather than textbook non-financial companies. In the traditional corporate framework, financial liabilities are only used for physical investment. Corporate data indicate that the financial assets and financial liabilities show a positive correlation in many large non-financial companies in China.

In contrast, financial assets and liabilities should be reversed under normal circumstances (for example, when financing projects, internal fund holdings decrease and external borrowings increase). Wang et al. (2015) also discussed substantial financial intermediary activities in non-financial listed companies in China. Du et al. (2017) found that non-financial listed companies in China and Central and Eastern European Transitional Economies (CEE) have such specific forms of “Borrow to Lend” shadow banking services in their operations.

Based on the existing literature, this paper conducts a systematic study on the “Borrow to Lend” shadow banking activities of non-financial listed companies in China and has contributed in three aspects. First of all, we clearly defined the meaning of “Borrow to Lend” and improved the identification method of “Borrow to Lend” based on the existing literature. Most of the previous literature used the correlation between financial assets and financial liabilities to identify enterprises’ “Borrow to Lend.” However, the activity does not consider the short-term cash management situation that the company may have. The increase in financial assets may be a short-term financial management behavior, which will eventually be transformed into the entity’s investment in the company. This article supplements a new identification method that uses the correlation between the increase in corporate debt and the increase in entity investment to identify the situation where the enterprise borrows from the bank but does not make entity investment, and this method further supports the enterprise’s “Borrow to Lend” activity. Secondly, based on existing methods and research, we added data about return on capital at the enterprise level and industry level to explore the relationship between the “Borrow to Lend” activities of non-financial listed companies and the return on capital, further supporting the existence of “Borrow to Lend” behavior. Finally, this article analyzes how monetary policy changes and the proportion of state-owned enterprise bank loans affect “Borrow to Lend” behavior, which is of great significance for the nation’s ability to manage financial risks, maintain credit fairness, and improve credit efficiency. In summary, based on the company’s financial data, this article studies the identification of “Borrow to Lend” from the perspectives of system and market, and the relationship between monetary policy and the proportion of state-owned enterprise bank loans and the “Borrow to Lend” activities of listed companies, through in-depth discussions about the micro-mechanism and macro-effects of credit fairness and efficiency.

## Data and Methodology

The financial statement data of Chinese-listed companies, the ownership attribute data of listed companies (to identify the ownership attributes of Chinese-listed companies and divide them into central enterprises, local state-owned enterprises, and private enterprises), and the data about financial markets such as China’s inter-bank repurchase and lending rates are all sourced from the China Stock Market and Accounting Research (CSMAR) database. The sample in this article only includes non-financial listed companies, focusing mainly on the possible “Borrow to Lend” activities of listed companies with relatively easy financing. The empirical analysis section of this article selects the quarterly data of China’s non-financial listed companies, including 118,541 observations of 3,553 companies, during a time span of 2007–2018. The continuous variable data in all samples are adjusted to the range of 1–99% to eliminate the influence of extreme values.

To estimate monetary policy shocks, we calculated China’s quarterly monetary policy shock variables based on the estimation method of identifying exogenous shocks (Chen et al., 2018). In addition, we use the data of the proportion of state-owned enterprise debt to the total debt of non-financial enterprises to measure the proportion of state-owned enterprise bank loans in China. The time span is from 2013 to 2018.

The data is from an International Monetary Fund (IMF) research report (the specific report is “Report on Article 4 Consultation between the Executive Board of the International Monetary Fund and China in 2019” https://www.imf.org/~/media/Files/Publications/CR/2019/Chinese/1CHNCA2019003.ashx).

These two macroeconomic variables assist us to explore how the “Borrow to Lend” activities of non-financial listed companies are affected by exogenous monetary policy shocks and the proportion of state-owned enterprise loans.

### Model Design and Assumptions

This article identifies strategy 1 of non-financial listed companies engaging in “Borrow to Lend” activities mainly based on the method of Shin and Zhao (2013). They examined the changes in financial assets and financial liabilities on the consolidated balance sheets of listed companies in China and the United States. Identification model 1 mainly observes the relationship between financial assets and financial liabilities. Short-term investments can also be liquidated quickly and roughly equivalent to cash; financial assets include cash and short-term investments in the following analysis. Since borrowing can also include long-term borrowing, financial liabilities are calculated as the sum of short-term borrowings and long-term borrowings.

To study the relationship between financial liabilities and financial assets, the model is as follows:


(1) $$F\,i\,n\,\_\,a\,s\,s_{i,t}=\beta *F\,i\,n\,\_\,l\,i\,a_{i,t}+\alpha *X_{i,t}+\gamma_{i}+\theta_{t}+\epsilon_{i,t}$$

In eq. (1), i and t refer to the company and year. The dependent variable *Fin*_*ass*_*i*,*t*_ in the above regression equation represents the financial assets of non-financial listed companies. The independent variable *Fin*_*lia*_*i*,*t*_ represents financial liabilities. *X*_*i,t*_ is the control variable, including Sales (measure the size of the enterprise), Leverages (measure the enterprise leverage), Tangibility (measure the tangible assets of the enterprise), ROE (measure the profitability of the enterprise), and TobinQ (measure the value of the enterprise). γ_*i*_ and θ_*t*_ represent firm and quarterly fixed effects. ε_*i*,*t*_ is the error term. According to the opinions of the previous literature and the actual situation, if β is not significantly negative, it indicates that the company has “Borrow to Lend” activities.

Model 2 is for identifying non-financial listed companies engaged in “Borrow to Lend” activities and is mainly based on the studies of Jiang et al. (2010); Wang et al. (2015). By examining the trend of accounts receivable and other issues in the consolidated balance sheet of non-financial listed companies, it identifies the credit intermediary activities of enterprises. The logic behind this is that once a company makes a loan, the inflow and outflow of funds will inevitably be recorded on the balance sheet and will be more concentrated in the total receivables and other receivable items. In addition, the notes to the financial statements of some companies show that inter-company loans are recorded in accounts receivable. Therefore, we believe that when accounts receivable and other accounts receivable and financial liabilities show positive changes, companies are more likely to have “Borrow to Lend” activities.

To study the relationship between accounts receivable and other accounts receivable and financial liabilities, we set up the model as follows:


(2) $$T\,o\,t\,\_\,r\,e\,c_{i,t}/O\,t\,h\,\_\,r\,e\,c_{i,t}=\beta *F\,i\,n\,\_\,l\,i\,a_{i,t}+\alpha *X_{i,t}+\gamma_{i}+\theta_{t}+\varepsilon_{i,t}$$

In eq. (2), the dependent variable *Tot*_*rec*_*i*,*t*_/*Oth*_*rec*_*i*,*t*_ represents the two accounting items of total accounts receivable and other accounts receivable, and the definition of other variables is the same as the variables in model (1). If β is significantly positive, it means that the company has “Borrow to Lend” activities from another perspective.

Model 3 identifies non-financial listed companies engaged in “Borrow to Lend” activities based on the literature about the decline in the investment rate of corporate entities. Zhang and Zhang (2016) discussed financialization’s direct or indirect impact on industrial investment. They found that economic financialization significantly reduced the industrial investment rate of enterprises. “Borrow to Lend” is a special manifestation of the financialization and shadow banking of non-financial listed companies. The decline in corporate investment growth is likely to be significantly related to “Borrow to Lend,” so here we will discuss the relationship between the increase in corporate borrowing and the increase in corporate investment of non-financial enterprises. As in normal business activities, the increase in corporate debt and the increase in investment should show a positive relationship; that is, after a company borrows from a bank or other financial institutions, the company will also increase investment to earn money from the perspective of borrowing costs, instead of leaving cash in their hands and increasing the cost of capital use or consuming the opportunity cost of investment. However, for abnormal non-financial companies, the increase in corporate borrowings or liabilities and the increase in investment have shown a reverse relationship; that is, the investment did not increase after corporate loans but declined, which confirms that there is the possibility of “Borrow to Lend” activities in non-financial companies from the side.

In the following analysis, we define the incremental investment △*Invest*_*i*,*t*_ as the sum of the increase in cash that the company invests in fixed assets, intangible assets, and other long-term assets and the increase in cash paid for the investment, and use the company’s sales for standardization (Jin et al., 2012). The increase in liabilities △*Fin*_*lia*_*i*,*t*_ is defined as the sum of the increase in short-term borrowings and long-term borrowings, and uses the company’s sales for standardization. To study the relationship between the increase in corporate financial liabilities and the increase in its investment, we set up the model as follows:


(3) $$△\,I\,n\,v\,e\,s\,t_{i,t+1}=\beta *△\,F\,i\,n\,\_\,l\,i\,a_{i,t}+\alpha *X_{i,t}+\gamma_{i}+\theta_{t}+\varepsilon_{i,t}$$

In eq. (3), we focus on the relationship between the increase in corporate investment △*Invest*_*i*,*t* + 1_ as the dependent variable and the increase in corporate financial liabilities △*Fin*_*lia*_*i*,*t*_ as the core explanatory variable; the other control variables are the same as in model (1). If β is significantly negative, it indicates that the company has borrowed money but has not used it for investment, and it is speculated that there is a “Borrow to Lend” activity. It is worth mentioning that the previous two “Borrow to Lend” identification strategies did not reflect the consideration of corporate cash management. If the company borrows money for short-term financial investment and then uses it for physical investment, this situation will also show a short-term positive correlation between financial assets and financial liabilities. Still, this should not be recognized as a “Borrow to Lend” activity. Therefore, model 3’s discussion of the negative correlation between the increase in corporate liabilities and the increase in corporate investment is important supplementary evidence to the first two identification models. We also take a one-period lag term for the financial liabilities on the right side of eq. (3) to determine that the financial liabilities in the current period have not been converted into entity investments in the future. This supports better identification of “Borrow to Lend” activities. As such, we put forward the first hypothesis of this article:

Hypothesis 1: If there is a negative relationship between the increase of financial liabilities of non-financial listed companies and the value-added of their investments, it can be considered that the company has “Borrow to Lend” activities.

Next, we will focus on analyzing how factors at different levels affect the “Borrow to Lend” activities of non-financial listed companies; that is, observing the impact of the internal characteristics of enterprises, industry characteristics, monetary policy, and changes in the proportion of state-owned enterprise loans on “Borrow to Lend” activities.

To study the impact of corporate characteristics and industry factors on “Borrow to Lend,” we will use model (1) as the basis and set the following models (4) and (5) as shown:


(4) $$F\,i\,n\,\_\,a\,s\,s_{i,t}=\beta_{1}*F\,i\,n\,\_\,l\,i\,a_{i,t}*F\,a\,c\,t\,o\,r\,s_{i,t}+\beta_{2}*F\,i\,n\,\_\,l\,i\,a_{i,t}+\alpha *X_{i,t}+\gamma_{i}+\theta_{t}+\varepsilon_{i,t}$$

(5) $$F\,i\,n\,\_\,a\,s\,s_{i,t}=\beta_{1}*F\,i\,n\,\_\,l\,i\,a_{i,t}*F\,a\,c\,t\,o\,r\,s_{I,t}+\beta_{2}*F\,i\,n\,\_\,l\,i\,a_{i,t}+\alpha *X_{i,t}+\gamma_{i}+\theta_{t}+\varepsilon_{i,t}$$

Of these, model (4) is the regression equation of the influence of enterprise-level influencing factors on enterprises’ “Borrow to Lend” activities. Among them, *Factors*_*i*,*t*_ are the influencing factors of enterprise-level, including Age (city age), Growth (main business income growth rate), and Rk (main business return rate), and the other control variables are the same as in model (1). Model (5) is the regression equation of the influence of industry-level influencing factors on the “Borrow to Lend” activities of enterprises. Among them, *Factors*_*I*,*t*_ are the industry-level influencing factors, including the average Intangible (intangible assets) and Rk (return on main business) of each industry, and the other control variables are the same as in model (1). Here we refer to the indicators of the rate of return on the main business as per the research of Zhang and Zhang (2016).

**FIGURE 1:**
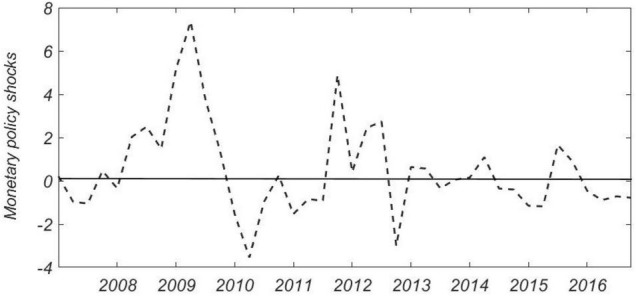
The exogenous shock component of monetary policy.

Based on the previous sections, we studied how the two types of macro-indicators (the impact of exogenous monetary policy and the proportion of state-owned enterprises in non-financial corporate bank loans) affect the “Borrow to Lend” activities of non-financial listed companies. Monetary policy consists of the expected endogenous growth of the money supply and the unexpected external growth of the money supply. To analyze the impact of monetary policy on enterprises’ “Borrow to Lend” activities, it is necessary to decompose the variable M2 that reflects the monetary policy in time series and separate the unanticipated exogenous part of the money supply. Based on Chen et al. (2018), this paper estimates an asymmetric monetary policy rule in line with China’s national conditions. It uses the time series method provided in the article to isolate the exogenous impact of China’s monetary policy as a measure of monetary policy tightness (see Figure 1). When the value is positive, it corresponds to a loose monetary policy; while the value is negative, it is considered to correspond to a tight monetary policy.

We analyze model (1) to explore how exogenous monetary policy shocks affect listed companies’ “Borrow to Lend” activities. At the same time, we also divide non-financial listed companies into state-owned enterprises and private enterprises, and design models respectively (6):


(6) $${\text{Fin\_ass}}_{i,t}=\beta_{1}*{\text{Fin\_lia}}_{i,t}*\text{Mp}+\beta_{2}*{\text{Fin\_lia}}_{i,t}+\alpha *{\text{X}}_{i,t}+\gamma_{\text{i}}+\theta_{\text{t}}+\varepsilon_{i,t}$$

In model (6), Mp is the exogenous impact of China’s monetary policy, which is quarterly data. The remaining control variables are the same as in eq. (1). Among them, coefficient β_1_ is our focus; that is, how the monetary policy Mp affects the secondary credit activities of non-financial listed companies. In addition, we also used 2008 and 2013 as time markers to compare the different situations after the financial crisis and after the implementation of the Basel III Agreement to test the difference in the impact of monetary policy on “Borrow to Lend” activities.

Previous studies have found that shadow banking activities such as entrusted loans have an overall negative relationship with the scale of formal credit, meaning entrusted loans may have an alternative role for formal credit when monetary policy tightens and formal credit tightens. However, the “Borrow to Lend” phenomenon that this article focuses on differs from the traditional shadow banking activities of entrusted loans. As the “Borrow to Lend” funds that this article focuses on come from banks and other financial institutions, after the monetary policy is tightened, the source of funds—the channels of primary distribution—is blocked. Therefore, enterprises’ “Borrow to Lend” scale will also decline, so the “Borrow to Lend” activities and monetary policy changes should positively correlate.

Hypothesis 2: “Borrow to Lend” and monetary policy present a positive relationship; that is, when monetary policy is tightened, the scale of “Borrow to Lend” activities of listed companies will decline. Listed companies are more likely to conduct “Borrow to Lend” activities when relaxed monetary policy.

This article also analyzes the correlation between “Borrow to Lend” and changes in the proportion of state-owned enterprise loans in all enterprise loans. In November 2010, the G20 Seoul Summit approved “Basel III” (Basel III), drafted by the Basel Committee, which established new capital and liquidity supervision standards in the banking industry and required the adherence of member states. It was to be implemented in 2013, and the requirements were to be fully met by 2019. On January 1, 2013, China began to implement the “Commercial Bank Capital Management Measures (for Trial Implementation),” known as the Chinese version of Basel III. To observe the impact on the “Borrow to Lend” activities of changes in the proportion of state-owned enterprise loans after implementing the Basel III Agreement, we used the data in an International Monetary Fund report. The data indicate that after 2013, the credit targets of Chinese commercial banks shifted significantly to state-owned enterprises, which may make loans for small/medium-sized private enterprises more difficult. We use eq. (7) to analyze the influence of the proportion of state-owned enterprise bank loans *SOE*_*Loan* on “Borrow to Lend” activities:


(7) $$F\,i\,n\,\_\,a\,s\,s_{i,t}=\beta_{1}*F\,i\,n\,\_\,l\,i\,a_{i,t}*S\,O\,E\,\_\,L\,o\,a\,n+\beta_{2}*F\,i\,n\,\_\,l\,i\,a_{i,t}+\alpha *X_{i,t}+\gamma_{i}+\theta_{t}+\varepsilon_{i,t}$$

In model (7), *SOE*_*Loan* is the proportion of state-owned enterprise bank loans in each quarter, and the remaining control variables are the same as in eq. (1). Among them, coefficient β_1_ is the main focus, reflecting the changes in the “Borrow to Lend” activities of non-financial listed companies under the influence of changes in the proportion of state-owned enterprise bank loans *SOE*_*Loan*.

Hypothesis 3: “Borrow to Lend” and the proportion of state-owned enterprise loans show a positive relationship; that is, when the proportion of state-owned enterprise loans increases, the scale of “Borrow to Lend” activities of listed companies will increase.

In summary, we have obtained the definition and calculation methods of all variables involved in the regression model and the results are outlined in the appendix (see Appendix Table A1).

Table 1 presents the descriptive statistical results of the main variables. Due to some listed companies’ lack of financial data during the sample period, the number of observations for each variable may differ.

**TABLE 1:** Descriptive statistics of main variables.

Variables	Observation	Mean	Median	Standard deviation	25%Qtr	75%Qtr
Fin_ass	89,950	−0.649	−0.716	1.283	−1.481	0.127
Fin_lia	87,335	−0.68	−0.559	1.661	−1.526	0.322
Oth_rec	115,472	−3.372	−3.415	1.762	−4.458	−2.336
Tot_rec	114,415	−1.465	−1.231	1.637	−2.204	−0.429
ΔFin_lia	97,982	−6.273	0	866.9	−0.0309	0.06
ΔInvest	99,946	−0.874	0.0197	207.9	−0.00345	0.087
Sales	115,787	20.53	20.50	1.73	19.5	21.56
Leverage	117,941	0.546	0.440	6.339	0.267	0.612
Tangibility	108,893	5.622	1.853	357.7	1.016	3.222
ROE	115,906	0.0822	0.0354	6.920	0.0108	0.075
TobinQ	108,921	5.354	1.704	357.6	0.945	2.947
Age	113,437	15.14	15	7.193	9	22
Intangible	117,690	0.0478	0.0317	0.0657	0.0141	0.0571
Growth	111,938	2.697	0.479	463.0	0.188	0.869

## Empirical Analysis

### Analysis of Secondary Credit Identification

This paper uses the panel fixed effects model to conduct empirical tests on the “Borrow to Lend” identification and related models. Table 2 presents the regression between financial liabilities (Fin_lia) and financial assets (Fin_ass) based on the recognition model 1. The first column in the table provides the regression results of the full sample of non-financial listed companies. The second and third columns comprise the regression results of state-owned and private listed companies, respectively. It is evident that the coefficients of financial liabilities (Fin_lia) and financial assets (Fin_ass) are 0.0607 and 0.118, respectively, which are all significant at the 1% statistical level. From the perspective of different enterprise ownership, the positive correlation between state-owned listed companies’ financial assets and financial liabilities is stronger than that of private enterprises, indicating that they are more likely to participate in “Borrow to Lend” activities. This is consistent with the current credit rationing status of China’s financial market. Looking at different year intervals, we found that the coefficient was the highest in 2016–2018, which may be related to the country’s deleveraging policy, indicating that the deleveraging policy did not affect listed companies but might instead cause private SMEs that were discriminated against in financing to borrow through secondary credit.

**TABLE 2:** Secondary credit identification model 1.

	(1)	(2)	(3)	(4)	(5)	(6)
Dependent:Fin_ass	Total samples	State–owned	Private	2009–2012	2013–2015	2016–2018
Fin_lia	0.0607***	0.118***	0.0540***	0.0899***	0.0547***	0.152***
	(0.00323)	(0.00479)	(0.00464)	(0.00580)	(0.00567)	(0.0112)
Sales	−0.354***	−0.331***	−0.348***	−0.443***	−0.575***	−0.487***
	(0.00499)	(0.00728)	(0.00712)	(0.00980)	(0.0112)	(0.0197)
Leverage	−0.0841***	−0.901***	−0.0708***	−0.0720***	−0.141***	−1.288***
	(0.00664)	(0.0294)	(0.00774)	(0.0141)	(0.0116)	(0.0756)
Tangibility	−0.0857***	−0.0766***	−0.0764***	−0.111***	–0.00593	–0.00577
	(0.00475)	(0.0115)	(0.00567)	(0.00998)	(0.00652)	(0.0130)
ROE	–0.000558	–0.00392	0.00110	–0.00296	–0.000922	0.00564
	(0.00224)	(0.00335)	(0.00299)	(0.00243)	(0.00451)	(0.00347)
TobinQ	0.0916***	0.0648***	0.0826***	0.117***	−0.0264***	−0.0484***
	(0.00508)	(0.0127)	(0.00600)	(0.0107)	(0.00761)	(0.0154)
Constant	6.646***	6.684***	6.476***	8.485***	11.37***	10.55***
	(0.103)	(0.154)	(0.144)	(0.200)	(0.232)	(0.412)
Observation	66,362	30,350	33,889	24,570	22,529	8,996
R2	0.721	0.762	0.689	0.837	0.845	0.903
Individual fixed effect	Control	Control	Control	Control	Control	Control
Time fixed effect	Control	Control	Control	Control	Control	Control

Standard errors are in parentheses. *** indicate significance at the statistical level of 10%.

To further test the heterogeneity and robustness of the “Borrow to Lend” activities of listed companies, we distinguish between local state-owned enterprises and central state-owned enterprises in state-owned enterprises and divide them into large and small enterprises according to the total assets of the listed companies. If the total assets are higher than the median of the same industry in the same year, they are regarded as large enterprises; otherwise, they are classified as small enterprises. The regression results are shown in Table 3. The first and second columns in Table 3 are divided into large and small central enterprises. The results show that the coefficients of financial liabilities (Fin_lia) and financial assets (Fin_ass) of central state-owned enterprises are both at 1%. The statistical level is significantly positive, and the coefficient is higher in large central state-owned enterprises, indicating that their “Borrow to Lend” activities are more active. The empirical results show that the coefficients of financial liabilities (Fin_lia) and financial assets (Fin_ass) of local state-owned enterprises are both significantly positive at the statistical level of 1%, with different scales. Significant levels of “Borrow to Len” activities of local state-owned enterprises are almost similar. The fifth and sixth columns in the table report the regression results of listed private companies participating in “Borrow to Lend” activities under different scales. It is apparent that “Borrow to Lend” activities are more prominent in large listed private companies but not in small private companies. This is because large listed private companies are usually a relatively important economic pillar. The close relationship between banks and enterprises in the local area allows them to avoid financing constraints caused by ownership discrimination as much as possible. Therefore, it is not difficult for these listed private companies to obtain financing. It is also possible for such companies to carry out financial activities in the form of “Borrow to Lend” activities, while small private enterprises do not experience these conditions.

**TABLE 3:** “Borrow to Lend” identification model 1: Enterprise heterogeneity.

	(1)	(2)	(3)	(4)	(5)	(6)
Dependent:Fin_ass	Large central enterprises	Small central enterprises	Large state-owned enterprises	Large state-owned enterprises	Large private enterprises	Small private enterprises
Fin_lia	0.211***	0.0814***	0.0910***	0.0939***	0.156***	0.00126
	(0.0486)	(0.0150)	(0.0126)	(0.00611)	(0.0119)	(0.00557)
Sales	−0.237***	−0.348***	−0.476***	−0.415***	−0.421***	−0.532***
	(0.0792)	(0.0225)	(0.0199)	(0.00992)	(0.0175)	(0.0102)
Leverage	−0.588*	−0.925***	0.222**	−0.890***	−0.938***	−0.0823***
	(0.316)	(0.107)	(0.111)	(0.0357)	(0.0922)	(0.00816)
Tangibility	2.511***	–0.125	−0.0485*	−0.102***	−0.0489**	−0.0574***
	(0.657)	(0.0816)	(0.0293)	(0.0137)	(0.0206)	(0.00624)
ROE	0.0983	0.0192*	0.0520**	−0.00652*	0.0122	–0.000848
	(0.127)	(0.0110)	(0.0236)	(0.00369)	(0.0182)	(0.00309)
TobinQ	−2.629***	0.0570	0.0914***	0.0942***	0.0402	0.0661***
	(0.722)	(0.0850)	(0.0352)	(0.0151)	(0.0251)	(0.00653)
Constant	5.091***	7.117***	9.872***	8.171***	9.016***	9.924***
	(1.931)	(0.475)	(0.442)	(0.204)	(0.369)	(0.200)
Observation	626	3,019	4,835	19,830	7,224	22,660
R2	0.901	0.754	0.886	0.745	0.824	0.713
Individual fixed effects	Control	Control	Control	Control	Control	Control
Time fixed effects	Control	Control	Control	Control	Control	Control

*, **, and *** indicate significance at the statistical level of 1, 5, and 10% respectively.

To further explore the “Borrow to Lend” activities of enterprises from different angles, Table 4 is based on the regression results of financial liabilities (Fin_lia) obtained from identification model 2 with enterprise accounts receivable (Tot_rec) and other accounts receivable (Oth_rec). Basis Identification model 3 examines the relationship between the increase in corporate financial liabilities (ΔFin_lia) and the increase in future entity investment (ΔInvest). In actual corporate activities, the differences in the accounts receivable between companies can reflect the differences in the scale of loans between companies to a greater extent. In addition, some companies will record the loan items as other receivable items when they are engaged in lending. It is necessary to reflect on the “Borrow to Lend” behavior of non-financial enterprises by analyzing accounts receivable. Columns 1 and 2 in Table 4 show the results of the relationship between financial liabilities and accounts receivable in the full sample, while columns 3 and 4 are the results of the relationship between financial liabilities and other accounts receivable in the full sample. The results suggest that regardless of the initial regression of the full sample of companies, or after adding relevant control variables, the relationship between corporate accounts receivable and other accounts receivable and financial liabilities are significantly positively correlated and stable. During normal business operations, the relationship between receivables and other receivables and financial liabilities is not clear; but when “Borrow to Lend” activities generally occur, part of the funds raised from financial liabilities will be included as corporate loans and in accounts receivable, so we observe a significant positive correlation. This result is consistent with the result of model 1, and both directly reflect the existence of the “Borrow to Lend” behavior of enterprises from the perspective of borrowing and lending.

**TABLE 4:** “Borrow to Lend” identification models 2 and 3.

	(1)	(2)	(3)	(4)	(5)	(6)
Dependent	Tot_rec	Tot_rec	Oth_rec	Oth_rec	Δ Invest	Δ Invest
Fin_lia	0.133***	0.0983***	0.265***	0.207***		
	(0.00254)	(0.00274)	(0.00375)	(0.00405)		
ΔFin_lia					−0.0157***	−0.0185***
					(0.00596)	(0.00637)
Sales		−0.229***		−0.331***		0.0180***
		(0.00412)		(0.00602)		(0.00325)
Leverage		−0.0136**		0.0727***		0.00161
		(0.00582)		(0.00723)		(0.00244)
Tangibility		–0.000395		0.0147**		0.00122
		(0.00421)		(0.00578)		(0.00129)
ROE		−0.00273*		−0.00641***		6.90e–05
		(0.00140)		(0.00211)		(0.000514)
TobinQ		−0.0320***		−0.0368***		–0.00110
		(0.00486)		(0.00607)		(0.00132)
Constant	−1.376***	3.439***	−3.112***	3.721***	−0.0739***	−0.448***
	(0.00260)	(0.0861)	(0.00381)	(0.125)	(0.00167)	(0.0673)
Observation	83,596	76,985	84,237	77,622	88,206	81,656
R2	0.850	0.861	0.723	0.737	0.339	0.344
Individual fixed effects	Control	Control	Control	Control	Control	Control
Time fixed effects	Control	Control	Control	Control	Control	Control

*, **, and *** indicate significance at the statistical level of 1, 5, and 10% respectively.

Columns 5 and 6 in Table 4 report the results of the identification model 3, which tests the relationship between the increase in corporate financial liabilities (ΔFin_lia) and the increase in future entity investment (ΔInvest). In the time dimension, we shifted the increase in the dependent variable entity investment relative to the increase in financial liabilities for a period in the future to control for the possibility of purchasing short-term financial products first and then investing in entities. The empirical results demonstrate that the increase in borrowing or liabilities of non-financial listed companies with “Borrow to Lend” activities and their future investment increases may establish a negative relationship; that is, after corporate loans, physical investment does not increase. Still, decreases can be used as important supplementary evidence. It supports the possibility of “Borrow to Lend” activities for non-financial enterprises.

To further test the heterogeneity and robustness of the “Borrow to Lend” activities of listed companies, we divide listed companies into state-owned enterprises and private enterprises. Table 5 examines the sub-sample regression results of identification models 2 and 3. Among them, the regression results of financial liabilities (Fin_lia) and corporate accounts receivable (Tot_rec) obtained in the first and second columns of Table 5 show that the correlation coefficients between state-owned enterprises and private enterprises are both significantly at the 1% statistical level. Positively, the coefficient is higher in state-owned enterprises, indicating that their “Borrow to Lend” activities are more active. Columns 3 and 4 are the regression results of financial liabilities (Fin_lia) and other accounts receivable (Oth_rec), respectively. The empirical results also conclude that state-owned enterprises are more active in “Borrow to Lend” activities. In addition, the fifth and sixth columns in the table test the heterogeneity of the relationship between the increase in financial liabilities of state-owned enterprises and private enterprises (ΔFin_lia) and the increase in future entity investment (ΔInvest). The results report no positive correlation between the increase of financial liabilities of non-financial listed companies and the increase of their investments. Instead, they show a significant negative correlation. This relationship is significant in the sub-sample of state-owned enterprises. It is not significant in the sub-sample of private enterprises, which indicates that state-owned enterprises spend less new borrowing on physical investment expenditures.

**TABLE 5:** “Borrow to Lend” identification models 2 and 3: Enterprise heterogeneity.

	(1)	(2)	(3)	(4)	(5)	(6)
	State-owned enterprises	Private enterprises	State-owned enterprises	Private enterprises	State-owned enterprises	Private enterprises
Dependent	Tot_rec	Tot_rec	Oth_rec	Oth_rec	ΔInvest	ΔInvest
Fin_lia	0.109***	0.0806***	0.197***	0.170***		
	(0.00458)	(0.00365)	(0.00550)	(0.00660)		
ΔFin_lia					−0.0471***	–0.000403
					(0.00894)	(0.00927)
Sales	−0.273***	−0.206***	−0.364***	−0.361***	0.0245***	0.0165***
	(0.00673)	(0.00542)	(0.00815)	(0.00966)	(0.00526)	(0.00447)
Leverage	0.0864***	−0.0233***	0.0320***	0.608***	0.000385	0.00144
	(0.0276)	(0.00597)	(0.00787)	(0.0398)	(0.00344)	(0.00352)
Tangibility	0.00710	–0.000436	0.0292***	−0.0737***	0.000463	0.00102
	(0.0114)	(0.00464)	(0.00652)	(0.0159)	(0.00647)	(0.00144)
ROE	–0.000597	−0.00477**	–0.00497	−0.00692**	−5.16e–05	0.000796
	(0.00192)	(0.00205)	(0.00315)	(0.00279)	(0.000524)	(0.00176)
TobinQ	−0.0528***	−0.0241***	−0.0423***	0.0247	0.00292	–0.000922
	(0.0127)	(0.00543)	(0.00673)	(0.0177)	(0.00744)	(0.00146)
Constant	4.153***	3.118***	4.340***	4.138***	−0.598***	−0.410***
	(0.143)	(0.111)	(0.166)	(0.205)	(0.112)	(0.0906)
Observation	34,401	40,278	34,890	40,386	34,795	44,322
R2	0.860	0.853	0.738	0.739	0.322	0.360
Individual fixed effects	Control	Control	Control	Control	Control	Control
Time fixed effects	Control	Control	Control	Control	Control	Control

** and *** indicate significance at the statistical level of 5, and 10% respectively.

On the whole, “Borrow to Lend” activities are common for both state-owned and private enterprises but are performed more prominently by state-owned enterprises. The results of the above three models confirm each other, and all point to the same results. Having given the empirical results of the “Borrow to Lend” identification strategy, we will now continue to analyze the impact of the three different dimensions of corporate characteristics, industry trends, and macro-environment on a company’s participation in “Borrow to Lend” activities.

### Analysis of Impact Mechanism

Financial assets (Fin_ass) are used as dependent variables in this paper. The market age (Age), main business income growth rate (Growth), and the intersection of main business yield (Rk) and financial liabilities (Fin_lia) are used as independent variables to analyze the influence of individual characteristics on the relevance of financial assets and financial liabilities, thus discussing the influence mechanism of different enterprise individual characteristics on “Borrow to Lend” activities. The empirical results are shown in Table 6.

**TABLE 6:** Influencing factors and “Borrow to Lend” activities at the enterprise level.

	Total sample	Growth		Profit	
Dependent:Fin_ass	Age	State-owned enterprises	Private enterprises	State-owned enterprises	Private enterprises
Fin_lia	−0.0423***	0.0510***	0.114***	0.127***	0.0539***
	(0.00731)	(0.00468)	(0.00485)	(0.00485)	(0.00470)
Fin_lia × Age	0.00618***				
	(0.000392)				
Fin_lia × Growth		-3.60e–05***	8.61e–05		
		(6.47e–06)	(0.000199)		
Fin_lia × Rk				−0.373***	0.00153
				(0.0341)	(0.0251)
Sales	−0.346***	−0.345***	−0.335***	−0.332***	−0.348***
	(0.00502)	(0.00720)	(0.00737)	(0.00727)	(0.00712)
Leverage	−0.0883***	−0.0638***	−0.884***	−0.891***	−0.0708***
	(0.00663)	(0.00771)	(0.0297)	(0.0294)	(0.00775)
Tangibility	−0.0869***	−0.0662***	−0.0706***	−0.0802***	−0.0764***
	(0.00479)	(0.00563)	(0.0116)	(0.0115)	(0.00567)
ROE	–0.000655	0.00107	–0.00398	–0.00454	0.00110
	(0.00224)	(0.00294)	(0.00331)	(0.00334)	(0.00299)
TobinQ	0.0936***	0.0714***	0.0567***	0.0680***	0.0826***
	(0.00512)	(0.00596)	(0.0128)	(0.0127)	(0.00601)
Constant	6.481***	6.401***	6.755***	6.705***	6.476***
	(0.104)	(0.146)	(0.156)	(0.154)	(0.144)
Observation	65,742	29,507	32,762	30,350	33,889
R2	0.722	0.697	0.767	0.762	0.689
Individual fixed effects	Control	Control	Control	Control	Control
Time fixed effects	Control	Control	Control	Control	Control

*** indicates significance at the statistical level of 10%.

First of all, the age of the entire sample of companies and “Borrow to Lend” activities show positive changes between each other, indicating that the longer a company’s listing time is, the more it will participate in “Borrow to Lend” activities. The companies that have been listed for a long time are generally state-owned enterprises or large-scale enterprises. Private enterprises with ownership advantages and high corporate goodwill can more easily obtain low-interest loans from banks and conduct “Borrow to Lend” activities, earning profit with interest margins. To further test the heterogeneity of the “Borrow to Lend” activities of listed companies, we divided them into state-owned enterprises and private enterprises for analysis when studying the growth and profitability of companies. The results show that the state-owned companies with poor growth will participate in more “Borrow to Lend” activities due to the slower development of their main business. In addition, the return on main business (Rk) of state-owned enterprises and their “Borrow to Lend” activities are significantly positive at the statistical level of 1%, indicating that the lower the return on the primary business of the enterprise, the more “Borrow to Lend” activities may be involved. Evidently, when there are bottlenecks or difficulties in their own development and profitability, state-owned enterprises may use their institutional advantages to obtain low-cost loans from banks for “Borrow to Lend” activities.

In contrast, the results for private enterprises are not significant as they do not exhibit such behavior. This result reflects the participation of enterprises with different ownerships in the “Borrow to Lend” activities. There are significant differences in the underlying mechanism of sub-credit.

Table 7 below shows the empirical test results of industry-level factors and corporate “Borrow to Lend” activities. First, we observed the relationship between the industry average intangible assets (Intangible) of the full sample and enterprises of different ownerships and enterprises’ “Borrow to Lend” activities. The results showed that the full sample and the intangible assets of state-owned enterprises have a significant negative correlation with the “Borrow to Lend” activities. The company’s intangible assets mainly include related assets such as patent rights, non-patent technologies, trademark rights, copyrights, and land use rights, which are the “soft power” of the company and the industry. The regression results show that the weaker the “soft power” of an industry, the higher the scale or level of “Borrow to Lend” enterprises’ activities. This situation mainly exists in state-owned enterprises. Some state-owned enterprises do not pay attention to the long-term development of scientific and technological innovation and have selected to shift financialization from the real to the virtual. Secondly, considering that the return on capital at the industry level is more exogenous than the operating activities of a single company, we analyzed the impact of the average return on main business (Rk) at the industry level on the “Borrow to Lend” activities of non-financial listed companies. The results in Table 7 reveal that in industries with low return on the main business, state-owned enterprises are more significantly involved in “Borrow to Lend,” which is consistent with the evidence of return at the enterprise level. This result further proves the relatively special economic and political status of state-owned enterprises in China. When their industry faces declining returns, falling behind, or even being eliminated, state-owned enterprises can still obtain loans from banks and conduct “Borrow to Lend” activities. Banking activities can earn interest margins to offset the decline in the physical industry.

**TABLE 7:** Industry-level influencing factors and “Borrow to Lend” activities.

Dependent:F*in*_a*ss*	Full sample	State-owned enterprises	Private enterprises	Full sample	State-owned enterprises	Private enterprises
Fin_lia	0.211*** (0.0157)	0.284*** (0.0223)	0.183*** (0.0243)	0.193*** (0.0116)	0.261*** (0.0174)	0.172*** (0.0148)
Fin_lia=Intangible	−0.378* (0.198)	−0.416* (0.234)	−0.245 (0.416)			
Fin_lia=Rk				−0.00147 (0.000952)	−0.0114*** (0.00341)	−0.00115 (0.000821)
Sales	−0.475*** (0.0164)	−0.417*** (0.0206)	−0.500*** (0.0228)	−0.475*** (0.0165)	−0.418*** (0.0206)	−0.500*** (0.0229)
Leverage	−0.111*** (0.0311)	−1.153*** (0.128)	−0.0994*** (0.0240)	−0.111*** (0.0310)	−1.154*** (0.128)	−0.0995*** (0.0240)
Tangibility	−0.0721*** (0.0139)	−0.0402 (0.0349)	−0.0736*** (0.0143)	−0.0724*** (0.0139)	−0.0386 (0.0350)	−0.0741*** (0.0141)
ROE	−0.00130 (0.00344)	−0.00730 (0.00467)	0.00147 (0.00372)	−0.00129 (0.00344)	−0.00735 (0.00466)	0.00149 (0.00371)
TobinQ	0.0801*** (0.0166)	0.0166 (0.0406)	0.0830*** (0.0158)	0.0804*** (0.0166)	0.0143 (0.0406)	0.0835*** (0.0157)
Observation	66,494	30,367	33,998	66,494	30,367	33,998
R2	0.387	0.427	0.373	0.386	0.426	0.373
Individual fixed effects	Control	Control	Control	Control	Control	Control
Time fixed effects	Control	Control	Control	Control	Control	Control

*, and *** indicate significance at the statistical level of 1 and 10% respectively.

### Analysis of Transmission Mechanism

To further explore the transmission mechanism of the “Borrow to Lend” of enterprises, we observe the impact of monetary policy and the ratio of state-owned enterprises in bank loans to all non-financial enterprises on the “Borrow to Lend” activities between enterprises from a more macro perspective. First, we use the Exogenous Monetary Policy Shock estimated by Chen et al. (2018) to measure the tightness of monetary policy, and explore the mechanism of influence of monetary policy on “Borrow to Lend” activities for companies in different periods.

Table 8 presents the changes in the “Borrow to Lend” activities of non-financial companies with the tightening of monetary policy. We divide the sample into the post-financial crisis period (from the second quarter of 2008 to the end of 2012) and the implementation period of the Chinese version of Basel III (from 2013 to the end of 2018), and observe whether there are variations in the correlation between different sample intervals. The first column represents the regression results of the full sample. After Crisis represents the regression results after the financial crisis and is divided into two sub-samples: state-owned enterprises and private enterprises. After Basel Event represents the regression results after the implementation of Basel III by Chinese commercial banks, and it is also divided into two sub-samples of state-owned enterprises and private enterprises.

**TABLE 8:** Monetary policy shocks and “Borrow to Lend” activities.

	(1)	After crisis		After Basel event	
Dependent:Fin_ass	Full sample	State-owned enterprises	Private enterprises	State-owned enterprises	Private enterprises
Fin_lia	0.0594***	0.106***	0.106***	0.116***	0.0413***
	(0.00331)	(0.00781)	(0.00789)	(0.00806)	(0.00633)
Fin_lia × Mp	0.000658**	0.00242**	0.00437***	0.00555*	−0.00995***
	(0.000869)	(0.00101)	(0.00133)	(0.00337)	(0.00366)
Sales	−0.358***	−0.443***	−0.379***	−0.461***	−0.501***
	(0.00522)	(0.0121)	(0.0133)	(0.0149)	(0.0117)
Leverage	−0.0764***	−0.549***	−0.0366***	−1.051***	−0.156***
	(0.00666)	(0.0494)	(0.0121)	(0.0575)	(0.0133)
Tangibility	−0.0851***	−0.0833***	−0.0967***	–0.0255	–0.00658
	(0.00478)	(0.0197)	(0.0122)	(0.0186)	(0.00623)
ROE	–0.00231	0.0240***	−0.00461*	−0.00988***	0.0110
	(0.00228)	(0.00807)	(0.00276)	(0.00295)	(0.00857)
TobinQ	0.0896***	0.0699***	0.0963***	–0.0152	−0.0239***
	(0.00511)	(0.0213)	(0.0126)	(0.0213)	(0.00725)
Constant	6.705***	8.738***	7.109***	9.742***	9.752***
	(0.107)	(0.253)	(0.266)	(0.315)	(0.239)
Observation	63,850	14,008	13,418	11,819	16,702
R2	0.724	0.829	0.806	0.853	0.792
Individual fixed effects	Control	Control	Control	Control	Control
Time fixed effects	Control	Control	Control	Control	Control

*, **, and *** indicate significance at the statistical level of 1, 5, and 10% respectively.

The regression results demonstrate that there is a significant positive correlation between the “Borrow to Lend” activities of non-financial enterprises and monetary policy (Mp); that is, when the monetary policy is loose, “Borrow to Lend” activities increase significantly and when the monetary policy is tightened, “Borrow to Lend” activities decrease significantly. Based on the sample interval, after the 2008 financial crisis, “Borrow to Lend” activities of state-owned enterprises and private enterprises are also significantly positively correlated with monetary policy. The stimulus policies adopted by the government after the financial crisis are more easily accessible. Non-financial listed companies with funds have a clear role in promoting “Borrow to Lend” activities. The reason is that banks are more inclined to give loans to listed companies as high-quality credit targets during the period of credit expansion. However, listed companies in the same period are usually key credit support objects. Therefore, listed companies may use “Borrow to Lend” activities to transfer excess credit to small/medium-sized private enterprises subject to financing constraints. This is different from the nature of entrusted loans in which enterprises use their own funds to lend to formal credit during the period of credit expansion. The “Borrow to Lend” activities between enterprises have supplemented and minimized the lack of credit for SMEs.

After the implementation of the Chinese version of Basel III in 2013, the coefficient of “Borrow to Lend” activities of state-owned enterprises and monetary policy was significantly positive at the statistical level of 10%; meaning, when monetary policy tends to tighten, the scale of “Borrow to Lend” in state-owned enterprises drops significantly (Sarfraz et al., 2020; Shah et al., 2021). The reason is that during the monetary policy tightening period, banks are restricted by the new version of the Basel Agreement, their lending assessment tends to be prudent, the overall lending scale has declined, and state-owned enterprises are also more cautious in conducting “Borrow to Lend” activities due to higher-level and internal regulatory requirements. The coefficient of “Borrow to Lend” activities and monetary policy of private listed companies is significantly negative at the 1% statistical level. That is, during the credit contraction period, listed private companies are less regulated than state-owned enterprises, and their actions can be compared with lending behavior. During the period of monetary expansion, the higher credit prices obtained short-term excess returns, and private enterprises played a more important role in “Borrow to Lend” activities when the monetary policy was tightened.

To further examine the transmission mechanism of “Borrow to Lend” activities of listed companies, we examined the relationship between “Borrow to Lend” and state-owned enterprise loans as a percentage of total non-financial corporate bank loans (SOE_Loan) from 2013 to 2018, and divided listed companies into a full sample of companies, state-owned listed companies, and private listed companies. Table 9 presents the empirical test result. The results indicate that the correlation coefficients of the full sample of companies, state-owned companies; and private companies are all significantly positive at the 1% statistical level. Therefore, as the proportion of state-owned enterprise bank loans in non-financial enterprises continues to increase, the “Borrow to Lend” activities of listed enterprises become more active. This shows that after the implementation of the Chinese version of Basel III in 2013, the credit targets of Chinese commercial banks began to shift significantly to state-owned enterprises, making loans for small and medium-sized private enterprises more difficult, and driving the necessity to borrow through “Borrow to Lend” activities.

**TABLE 9:** The proportion of state-owned enterprise loans and the “Borrow to Lend” activities of enterprises.

	(1)	(2)	(3)
Dependent:Fin_ass	Full sample	Listed state-owned enterprises	Listed private enterprises
Fin_lia	0.108***	0.150***	0.104***
	(0.00496)	(0.0083)	(0.00658)
Fin_lia × SOE_Loan	0.0128***	0.00730***	0.0163***
	(0.00121)	(0.00167)	(0.00171)
Sales	−0.693***	−0.663***	−0.697***
	(0.006)	(0.0099)	(0.00796)
Leverage	−0.234***	−1.092***	−0.193***
	(0.0147)	(0.0594)	(0.0162)
Tangibility	–0.00367	–0.0293	–0.00179
	(0.00574)	(0.0185)	(0.00665)
ROE	–0.00195	−0.00562*	0.00567
	(0.00317)	(0.0031)	(0.00802)
TobinQ	−0.0364***	–0.0313	−0.0347***
	(0.00666)	(0.0215)	(0.00765)
Constant	14.06***	14.21***	13.90***
	(0.124)	(0.204)	(0.161)
Observation	27,699	11,189	15,870
R2	0.813	0.856	0.787
Individual fixed effects	Control	Control	Control
Time fixed effects	Control	Control	Control

*, and *** indicate significance at the statistical level of 1 and 10% respectively.

To further prove the credibility of the empirical results of this paper, we conducted a related robustness test. We use the annual data of listed companies to study the identification and influence mechanism of “Borrow to Lend” activities and select the annual data of China’s non-financial listed companies, including 26,612 observations of 3,553 companies, during a time span of 2007–2018. The results show that the regression results of the identification model are still robust. In addition, the correlation between the “Borrow to Lend” activities of non-financial companies and the profitability of the company, monetary policy changes, and other variables is empirically tested. The regression results are robust and the research conclusions are relatively robust.

## Discussion and Conclusion

The institutional environment of uneven credit distribution in China provides a good research background for studying the “Borrow to Lend” activities of non-financial enterprises. Although the share of Chinese state-owned enterprises is declining year by year, state-owned enterprises still play an important role in the economy. The government’s implicit guarantee makes state-owned enterprises considered less risky when banks make loan decisions, and it is easier for them to obtain low-cost credit from banks, while small/medium-sized private enterprises are subject to double credit ration constraints from public ownership of the banking industry and the market. The financing differences in recent years have made it possible for state-owned enterprises to participate in “Borrow to Lend” shadow banking activities.

This paper uses the financial statement data of listed companies to identify the “Borrow to Lend” activities of non-financial listed companies, and adopts the residual of the exogenous M2 growth rate as a measure of monetary policy tightness, and the proportion of state-owned enterprises in non-financial corporate bank loans. It studies how monetary policy and the proportion of state-owned enterprise bank loans affect the “Borrow to Lend” activities of enterprises. The empirical results reveal that China’s non-financial listed companies conduct “Borrow to Lend” activities; that is, they borrow from banks and lend to other economic entities. In addition, monetary policy and the proportion of state-owned enterprise loans have an important influence on the “Borrow to Lend” activities of enterprises. The empirical results indicate that after the 2008 financial crisis, listed companies have participated in “Borrow to Lend” activities more significantly, but more stringent monetary policy will weaken the scale of “Borrow to Lend” activities. Due to different sources of funds, the findings of this article are different from the previous conclusions of the literature on entrusted loans. Further, it also demonstrates that it is necessary to distinguish between “Borrow to Lend” activities with bank credit as the source of funds and entrusted loans with self-owned funds as the main source. In terms of the proportion of state-owned enterprise loans, after the implementation of the Chinese version of Basel III in 2013, as the proportion of state-owned enterprise bank loans in non-financial companies continued to increase, the “Borrow to Lend” activities of listed companies became more active.

In general, the phenomenon of “Borrow to Lend” activities highlights the efficiency and fairness of credit distribution in China’s financial market. The large amount of funds borrowed by state-owned enterprises is used to arbitrage interest rates in different ways under the operation of “Borrow to Lend” activities. The cycle between similar financial institutions and large non-financial listed companies to obtain profits has caused serious “fund idling” and simultaneously aggravated financial risks. This study concludes that under the influence of loose monetary policy and the rising proportion of state-owned enterprise loans, non-financial listed companies have developed more serious economic problems such as “removal from reality to virtual” and “financial idling” of funds through “Borrow to Lend” activities. This makes credit lack efficiency and fairness in distribution, problems that require urgent attention from policy departments perfecto devise effective solutions. It is hoped that the relevant departments will curb the “Borrow to Lend” behavior of state-owned enterprises and large private enterprises and establish a long-term mechanism to solve the difficulty of financing for small, medium, and micro enterprises as soon as possible.

## Data Availability Statement

Publicly available datasets were analyzed in this study. These data can be found here: https://www.imf.org/~/media/Files/Publications/CR/2019/Chinese/1CHNCA2019003.a.

## Author Contributions

All authors listed have made a substantial, direct, and intellectual contribution to the work and approved it for publication.

## Conflict of Interest

The authors declare that the research was conducted in the absence of any commercial or financial relationships that could be construed as a potential conflict of interest.

## Publisher’s Note

All claims expressed in this article are solely those of the authors and do not necessarily represent those of their affiliated organizations, or those of the publisher, the editors and the reviewers. Any product that may be evaluated in this article, or claim that may be made by its manufacturer, is not guaranteed or endorsed by the publisher.

## Appendix

**APPENDIX TABLE A1:** Definition and calculation of main variables.

Variables	Definition	Variable calculation
Fin_ass	Financial assets	Divide the sum of cash holdings and short-term investments by its sales and take the logarithm
Fin_lia	Financial liabilities	Divide the sum of long-term and short-term loans by its sales and take the logarithm
Oth_rec	Other accounts receivable	Divide other accounts receivable by its sales and take the logarithm
Tot_rec	accounts receivable	Divide accounts receivable by its sales and take the logarithm
ΔFin_lia	Increase of financial liabilities	Value added/sales of long-term and short-term liabilities
ΔInvest	Increase of investment	(Increase in cash paid for fixed assets, intangible assets, and other long-term assets + increase in cash paid for investment)/Sales
Sales	sales	Natural logarithm of business sales
Leverage	Corporation’s leverage	Total corporate liabilities/total assets
Tangibility	Tangible assets ratio	Stock price/[(total assets-net intangible assets-net goodwill) ending value/ending value of paid-in capital]
ROE	Return on equity	Net profit/shareholders’ equity
TobinQ	Tobin Q	(Total market value of stocks + book value of debt)/book value of total assets
Age	Listing years	The year of the current year-the year the company went public
Intangible	Intangible assets	Intangible assets/total assets
Growth	growth	Main business income of the current year/Last year’s main business income-1
Rk	Main business rate of return	Main business rate of return = (operating income-operating costs-business taxes and surcharges-period expenses-asset impairment losses)/operating assets (i.e., operating capital + fixed assets + net value of long-term assets such as intangible assets)
SOE_Loan	Proportion of state-owned enterprise loans	Total loans of state-owned enterprises/total loans of non-financial enterprises
